# Lead Repositioning Guided by Both Physiology and Atlas Based Targeting in Tourette Deep Brain Stimulation

**DOI:** 10.5334/tohm.140

**Published:** 2020-07-08

**Authors:** Jackson N. Cagle, Wissam Deeb, Robert S. Eisinger, Rene Molina, Enrico Opri, Marshall T. Holland, Kelly D. Foote, Michael S. Okun, Aysegul Gunduz

**Affiliations:** 1 J. Crayton Pruitt Department of Biomedical Engineering, University of Florida, Gainesville, FL, US; 2 Department of Neurology, University of Florida Fixel Institute for Neurological Diseases, Gainesville, FL, US; 3 Department of Neuroscience, University of Florida, Gainesville, FL, US; 4 Department of Electrical and Computer Engineering, University of Florida, Gainesville, FL, US; 5 Department of Neurosurgery, University of Iowa, Iowa City, IA, US; 6 Department of Neurosurgery, University of Florida Fixel Institute for Neurological Diseases, Gainesville, FL, US

**Keywords:** Tourette syndrome, Deep brain stimulation, Electrophysiology, Centromedian thalamus, Brain mapping

## Abstract

**Background:**

The centromedian (CM) region of the thalamus is a common target for deep brain stimulation (DBS) treatment for Tourette Syndrome (TS). However, there are currently no standard microelectrode recording or macrostimulation methods to differentiate CM thalamus from other nearby structures and nuclei.

**Case Report:**

Here we present a case of failed conventional stereotactic targeting in TS DBS. Postoperative local field potential recordings (LFPs) showed features including beta power desynchronization during voluntary movement and thalamo-cortical phase amplitude coupling at rest. These findings suggested that the DBS lead was suboptimally placed in the ventral intermediate (VIM) nucleus of the thalamus rather than the intended CM region. Due to a lack of clinical improvement in tic severity scales three months following the initial surgery, the patient underwent lead revision surgery. Slight repositioning of the DBS leads resulted in a remarkably different clinical outcome. Afterwards, LFPs revealed less beta desynchronization and disappearance of the thalamo-cortical phase amplitude coupling. Follow-up clinical visits documented improvement of the patient’s global tic scores.

**Discussion:**

This case provides preliminary evidence that combining physiology with atlas based targeting may possibly enhance outcomes in some cases of Tourette DBS. A larger prospective study will be required to confirm these findings.

**Highlight:**

This report demonstrates a case of failed centromedian nucleus region deep brain stimulation (DBS). We observed suboptimal tic improvement several months following DBS surgery and subsequent lead revision improved the outcome. The neurophysiology provided an important clue suggesting the possibility of suboptimally placed DBS leads. Repeat LFPs during lead revision revealed less beta desynchronization and disappearance of the thalamo-cortical phase amplitude coupling. There was improvement in tic outcome following slight repositioning during bilateral DBS lead revision. This case provides preliminary evidence supporting the use of physiology to augment the atlas based targeting of Tourette DBS cases.

## Background

Gilles de la Tourette syndrome (TS) is a childhood onset neuropsychiatric disorder typified by both motor and phonic tics as well as comorbid psychiatric symptoms [5,6]. Deep brain stimulation (DBS) is currently an experimental treatment strategy for patients with severe and medication refractory TS [22,28,29]. Collective evidence drawn from neuroimaging [4,30,32], stereotactic lesions [1,10,21], and animal models [15,16,33] has demonstrated that the thalamo-striato-cortical circuit [17] is involved in the pathophysiology of TS [7]. The Centromedian (CM) nucleus region of the thalamus has been commonly targeted, but there are significant challenges in stereotactically targeting this area.

The optimal location for TS DBS has been at the center of debate over the past decade. The International Deep Brain Stimulation Database and Registry recently reported the clinical outcomes of 270 TS patients who underwent DBS treatment (2012 to 2019) across 10 countries. The CM thalamic region was the most common site of implantation (50.7%), followed by the anterior globus pallidus internus (28.1%) [27]. The CM nucleus region is difficult to define. It is one of over 30 subnuclei of the thalamus and is located in the ventroposterior region, measuring about 300 mm [3] in volume. Classical MRI image-based analysis must be coupled with a deformable atlas overlay, but even this approach may result in an ill-positioned CM nucleus DBS lead. Currently, no reliable functional mapping approach is available for targeting the CM nucleus, though this issue has become a potential area for research focus [23,31]. Furthermore, it has been recently shown that based on the DBS Registry and Database, there exists a large variability in the active DBS contact location within the CM thalamic region [11].

Here we report a single TS patient with failed stereotactic targeting in the CM nucleus region. We analyzed the neurophysiological signals obtained from the deep electrodes during post-operative visits and we also analyzed the post-operative brain imaging. Though we only moved the DBS lead a few millimeters, we observed different electrophysiological signal and also improvements in tic suppression.

## Case Report

### History and Examination

A 38 year old man presented to the Fixel Institute for Neurological Diseases for evaluation of medication refractory tics. His tics began at the age of 6 years and he was diagnosed with TS at the age of 12. He reported multiple different motor and phonic tics. His motor tics included head turning, bilateral shoulder shrugging, kicking of his legs (out to the side), swinging the right arm to the side, and neck jerking with associated eye rolling. His phonic tics included sniffing, throat clearing, grunting, animal noises, and coprolalia.

The patient’s Yale Global Tic Severity Scale (YGTSS) total score was 82 with an impairment subscale score of 40. His tics were disabling in social, occupational, and academic domains. His clinical presentation was complicated by co-morbid obsessive-compulsive disorder (OCD), attention-deficit hyperactivity disorder (ADHD), and depression. However, at the time of surgical implantation, his psychiatric comorbidities were optimized and he was on stable medication dosages. His OCD remained relatively severe, with a Yale-Brown Obsessive-Compulsive Scale (YBOCS) score of 26, and he was stable on his psychiatric treatment regimen (pre- and post-DBS) as summarized in Table 1. The medications were not changed before and after DBS implantation and there were no gaze disturbances, visual symptoms, or change in libido associated with the medications.

**Table 1:** Tourette Patient Medications.

Treatment at the time of DBS placement*	Prior treatment trials	Reason(s) for discontinuing medications

– **Amantadine 100 mg three times daily**	Haloperidol	Aggression and dystonia
	Pimozide	Dystonia
– **Atorvastatin 10 mg every night**	Risperidone	Dystonia
– **Guanfacine 1 mg every morning and 2 mg every evening**	Aripiprazole	Not effective even at 20mg daily dose
– **Olanzapine 20 mg every night**	Pergolide	Withdrawn from the market, psychosis
– **Paroxetine 20 mg every morning and 60 mg every evening**	Clonidine	Severe hypotension, lethargy, and drowsiness
	Benztropine	Blurry vision
	Clonazepam	Excessive drowsiness
	Topiramate	Not effective, dry mouth, and swelling in feet

* Medications did not change between the two surgeries discussed in this case report.

Prior to being evaluated at our center, he had tried multiple (unsuccessful) medication regimens for the management of his tics (Table 1) except VMAT-2 inhibitor, a common treatment for hyperkinetic disorders [18], because it was not required by the IRB protocols. These trials were either ineffective or produced side effects. He declined comprehensive behavioral intervention for tic therapy or habit reversal therapy and the IRB and FDA protocols did not require these therapies to be instituted prior to DBS. Following a multidisciplinary DBS evaluation, his TS was deemed severe, debilitating, and refractory to available treatment. His psychiatric comorbidities were stable and well controlled and he was referred for DBS evaluation.

### Surgery Procedure

The patient consented to be part of the National Institutes of Health (NIH) funded DBS in TS study (IRB201300850, NCT02056873). Subjects enrolled in the study underwent two stages of stereotactic surgical intervention [8]. During the first stage of the operation, Medtronic Model 3387 DBS leads were implanted bilaterally in the CM nuclei region. The electrode target trajectories for the CM nuclei were planned using direct visualization and manipulation of a modified digital Schaltenbrand-Bailey deformable atlas [26]. The atlas was morphed onto the patient’s pre-operative magnetic resonance imaging (MRI) FGATIR image [25] and co-registered to a pre-operative computed tomography image (CT) through the utilization of a Cosman-Roberts-Wells (CRW) stereotactic frame (as the reference). Medtronic RESUME II strip leads Model 3587A were placed along a stereotactially planned trajectory towards the hand primary motor cortex (M1). During the second stage of the operation approximately one month later, two Medtronic Activa PC+S neurostimulators (Medtronic PLC, Minneapolis, MN) were implanted in the bilateral subclavicular region and connected to the leads via extension wires. The Activa PC+S was not connected during the initial lead implantation surgery.

### Post-operative Follow-up

A post-operative CT image was obtained one month after the first stage DBS surgery to verify the location of the DBS electrodes. After the DBS surgery, the patient returned for six monthly post-surgical visits. During these visits, an experienced clinician programmer adjusted the stimulation parameters with the goal of optimization through tic reduction. Tic improvement was measured using the YGTSS scale each month. In addition to the clinical programming, local field potential (LFP) recordings were captured using the Activa PC+S system and were analyzed in MATLAB 2016a (Mathworks, Inc).

### Results

**Figure 1:**
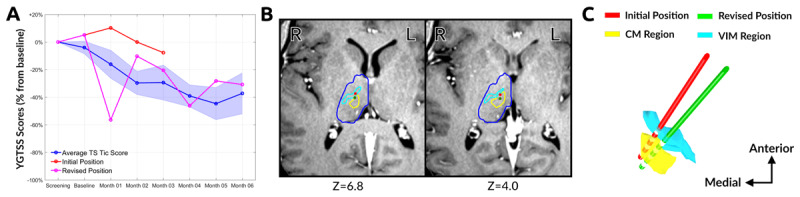
**A)** Yale Global Tic Severity Scale (YGTSS) of the patient prior to DBS surgery, the clinical outcome with initial lead location, and the clinical outcome after lead repositioning surgery. The tic scale was reduced by only 8% following the initial bilateral surgery and reduced by 30% post-repositioning. **B)** The patient’s T1-MRI in AC-PC coordinate space. The thalamus (blue outline), VIM nucleus (cyan outline), and CM nucleus region (yellow outline) are shown based on the modified digital Schaltenbrand-Bailey atlas. The red dot denotes the location of the electrode prior to repositioning and the green dot denotes the location of electrode following lead repositioning. The stereotactic coordinates of the original placement were: Anterior-Posterior (AP) –7.21 mm, Lateral (LT) 6.18 mm, and Axial (AX) 0.50 mm from midcommissural point with AC-PC plane entry angle of 54 and a central plane entry angle of 19. The stereotactic coordinate of the revised placement was: AP –9.77 mm, LT 5.57 mm, and AX –0.35 mm from the mid-commissural point with an AC-PC plane entry angle of 56 and a central plane entry angle of 27. **C)** The 3-D reconstruction of the atlas and DBS lead in its initial location and post-repositioning location is provided in a top-down view.

**Figure 2:**
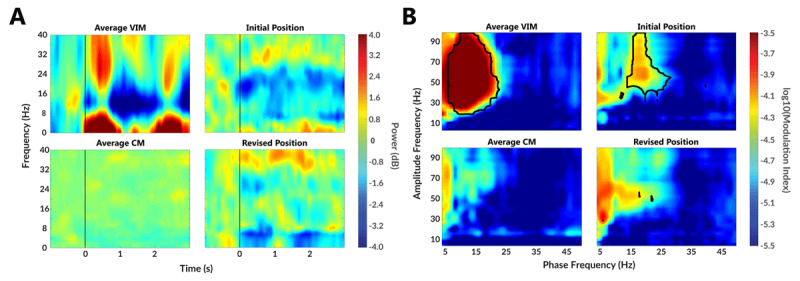
Comparison of neural features during voluntary movement. The average VIM spectrogram was collected in a previous ET study (conducted at UF Health) with leads implanted in the VIM thalamic nucleus (n = 2). Also, we provide data on the average CM spectrogram that was collected from other TS patients (n = 3) in the same study as the current case report. **A)** The spectrogram during voluntary movement with initial lead location shows strong beta desynchronization around the beta band (12Hz–30Hz), which disappears following the revision surgery. **B)** Prior to lead revision surgery, strong PAC was observed between the thalamic beta phase and cortical gamma (>40 Hz) amplitude, and this feature disappeared following revision surgery.

After three months of clinical programming, the patient’s tics had only improved by 8% (Figure 1A). Post-operative CT imaging revealed that the electrode was slightly anterior and superior compared to previous TS cases in the study (n = 3). The LFPs revealed beta band (12–30 Hz) power desynchronization with respect to movement onset during behavioral tasks (Figure 2A) and the power band remained desynchronized throughout the movement. This beta desynchronization is a common feature observed in the ventral intermediate nucleus (VIM) region. This region is both lateral and ventral to the CM region of the thalamus [3]. Similarly, a strong PAC between the depth electrode (phase) and the subdural strip (amplitude) was observed (Figure 2B). Malekmohammadi et al. [13] and our group have both shown the presence of PAC between the VIM and M1 [19]. This PAC was not a feature observed in the three other TS patients recruited into the study. These structural and functional results suggested that the initial lead was closer to the VIM rather than the CM region. The DBS interdisciplinary clinical team performed a careful risk-benefit analysis. The factors in the analysis included absence of clinically relevant tic suppression, subtle imaging differences from previous TS DBS cases, the patient’s initial post-operative imaging, and the use of physiologic data. These data were discussed with the patient and family and a decision was made to attempt repositioning the DBS leads.

The initial DBS placement and repositioning are summarized in Figure 1B using a digital deformable Schaltenbrand-Bailey atlas overlaid on the patient’s T1-MRI after transforming to AC-PC coordinates. The 3D reconstructed atlas, lead position, and lead trajectory are shown in Figure 1C. Following repositioning, there was a reduction in beta desynchronization and disappearance of the PAC between the depth electrodes and the subdural strips (Figure 2). There was also a greater “honeymoon” effect reported at the one month follow up visit following revision surgery. Following lead revision, identical postoperative monthly follow-up visits were conducted by the same clinician programmer. Tic scales were collected by the same rating psychiatrist and there was significant improvement in the clinical outcome (Figure 1A).

## Discussion

One of the challenges of targeting the CM region is the lack of an anatomical landmark and the lack of a standardized physiological approach to determine whether or not a lead is optimally placed. We know from the International Tourette DBS Registry and Database that many patients implanted fall below a 30% improvement threshold in motor and vocal tics [14]. We have no current methodology to determine the potential prevalence of suboptimal lead placement, however we suspect at least in some subset of cases that suboptimal benefit is tied to lead position. In this case report, we presented results of TS DBS performed in the same patient, in two anatomically close, but physiologically different thalamic nuclei. Slight repositioning of the DBS leads resulted in a remarkably different clinical outcome. The failure of significant tic improvement following initial lead placement generated a review of the patient’s postoperative imaging and a review of his associated physiology. We compared the patient’s physiological signal features to known physiology from the CM region in the other TS patients in our study. We also compared the physiology to the VIM nucleus region physiology from our essential tremor (ET) population. Previous intraoperative electrophysiology studies in TS have revealed increased low frequency activity, and also beta band modulation which was not present in the CM region [24]. Because the anatomical differences were subtle between the VIM and CM regions, the physiological data were critical in making the determination that the lead was in a suboptimal position.

In this patient several factors pointed to a suboptimally placed lead. First, beta desynchronization was observed during voluntary movement. Further, there was strong connectivity between the VIM and M1 cortex using PAC. Third, careful evaluation of the lead placement revealed the electrode to be in a slightly different position from the other TS patients enrolled in the study. Repositioning the depth electrode led to a reduction of signal features modulated by voluntary movements (i.e. the hallmark of VIM LFPs). The repositioning led to an emergence of potential CM signal features. This information collectively could be useful to other groups for initial targeting or for evaluation of previously implanted TS DBS patients.

This case of a suboptimally-placed lead also provides an important insight into the differing physiological signals in the VIM and CM nuclei regions of the thalamus and the potentially important role of the CM nucleus region in TS pathophysiology. The electrophysiology of CM and VIM nuclei presented in this paper may have future implications for surgical targeting of the CM region, especially for the use of adaptive DBS techniques which will require the presence of a reliable signal derived from the CM nucleus region. An intraoperative examination of TS electrophysiology could help to refine the CM region target location in the operating room and could potentially reduce the potential for a future revision surgery, but a larger prospective study is needed. Another alternative approach to using physiology could be the development of a refined image-based approach which could harness newer technologies to refine the final lead location (DTI, fMRI, etc.).

CM nucleus region thalamotomy for TS has yielded mixed results [20]. Individual reported outcomes have ranged from as low as no benefit to as high as 100% reduction in the YGTSS. Babel et al (2001) reported 12.5% to 75% combined motor/vocal tics scores reduction among 11 reported participants [1]. Balderman et al reported that 52.3% of TS CM thalamotomy patients achieved over 50% reduction of tics [2]. This result agrees with our recent image-based analysis of the International TS DBS Registry and Database, which revealed large variability in electrode placement across institutions [11]. The size of the thalamotomy ablation is generally larger than the current field in DBS, and we believe that improving the DBS targeting to more precise smaller region than thalamotomy can possibly improve Tourette DBS outcome with reduced side effects. A technique that combines image based and intraoperative physiology could have the potential to improve accuracy and clinical outcomes.

There are important limitations to this study. This is a single case report. Although the data has been consistent with prior experience in Tourette and tremor cases, it is possible that as more cases are reported, the results may be more nuanced. Second, the anatomy of thalamic subregions is extremely difficult to delineate on current MRI imaging and requires inferring locations via a deformable atlas. These atlases usually align only to visible structures. Despite these limitations, our findings confirm the important role of the CM nucleus region in TS and that stimulation within this region has beneficial effects on tics. In contrast, the VIM region may prove to be a less ideal target. Although our recent image-based analysis of the international registry was unable to identify an anatomical “hotspot” for optimal TS DBS, it is possible that the current analysis was limited by a lack of spatial specificity when performing group analysis after non-linear normalization; therefore, the region of stimulation in responders and non-responders could be intermixed [11]. Finally, tractography-based analyses have shown promising results and newer image based technologies (e.g. DTI) may offer an alternate or complementary approach to physiology [9,12].

In summary, the neurophysiology provided an important clue suggesting the possibility of suboptimally placed DBS leads. Repeat LFPs during lead revision revealed less beta desynchronization and disappearance of the thalamo-cortical phase amplitude coupling. There was improvement in tic outcome following slight repositioning. This case provides data to support the potential use of physiology to augment the current atlas based targeting of Tourette CM region DBS cases.

## Author Information

Michael S. Okun and Aysegul Gunduz are Authors.
